# Epidemiological Surveillance of HIV-1 Transmitted Drug Resistance in Spain in 2004-2012: Relevance of Transmission Clusters in the Propagation of Resistance Mutations

**DOI:** 10.1371/journal.pone.0125699

**Published:** 2015-05-26

**Authors:** Yolanda Vega, Elena Delgado, Aurora Fernández-García, Maria Teresa Cuevas, Michael M. Thomson, Vanessa Montero, Monica Sánchez, Ana Maria Sánchez, Lucia Pérez-Álvarez, Spanish Group for the Study of New HIV-1 Diagnoses in Galicia and Basque Country

**Affiliations:** 1 Unidad de Biología y Variabilidad de VIH, Centro Nacional de Microbiología, Instituto de Salud Carlos III, Majadahonda, Madrid, Spain; McGill University AIDS Centre, CANADA

## Abstract

Our objectives were to carry out an epidemiological surveillance study on transmitted drug resistance (TDR) among individuals newly diagnosed of HIV-1 infection during a nine year period in Spain and to assess the role of transmission clusters (TC) in the propagation of resistant strains. An overall of 1614 newly diagnosed individuals were included in the study from January 2004 through December 2012. Individuals come from two different Spanish regions: Galicia and the Basque Country. Resistance mutations to reverse transcriptase inhibitors (RTI) and protease inhibitors (PI) were analyzed according to mutations included in the surveillance drug-resistance mutations list updated in 2009. TC were defined as those comprising viruses from five or more individuals whose sequences clustered in maximum likelihood phylogenetic trees with a bootstrap value ≥90%. The overall prevalence of TDR to any drug was 9.9%: 4.9% to nucleoside RTIs (NRTIs), 3.6% to non-nucleoside RTIs (NNRTIs), and 2.7% to PIs. A significant decrease of TDR to NRTIs over time was observed [from 10% in 2004 to 2% in 2012 (p=0.01)]. Sixty eight (42.2%) of 161 sequences with TDR were included in 25 TC composed of 5 or more individuals. Of them, 9 clusters harbored TDR associated with high level resistance to antiretroviral drugs. T215D revertant mutation was transmitted in a large cluster comprising 25 individuals. The impact of epidemiological networks on TDR frequency may explain its persistence in newly diagnosed individuals. The knowledge of the populations involved in TC would facilitate the design of prevention programs and public health interventions.

## Introduction

The success of antiretroviral treatment may be limited by the emergence of HIV drug resistance, which can be transmitted to newly infected individuals. HIV transmitted drug resistance (TDR) is of public health concern because it has the potential to compromise the efficacy of antiretroviral therapy (ART) at the population level and can contribute to failure of first-line ART.

Studies on TDR carried out in different countries report TDR prevalences ranging between 0% and 27% [1,2]. Although the prevalence of TDR in Spain differs between regions and time periods, the reported overall rate is around 10% in treatment-naïve HIV-1 subtype B-infected patients [3–7].

To accurately compare TDR rates across geographic regions and times, the World Health Organization (WHO) has recommended the adoption of a consensus genotypic definition of transmitted HIV-1 drug resistance [8]. For this, surveillance drug-resistance mutations (SDRM) were selected for their suitability as indicators of transmitted resistance. The criteria for their selection were that these mutations are commonly recognized as causing or contributing to resistance, are nonpolymorphic in untreated persons, and are applicable to all HIV-1 subtypes [8,9]. A standard list of SDRMs makes it possible to compare the prevalence of transmitted resistance at different times and regions and to facilitate meta-analyses of surveillance data collected by different groups. WHO TDR surveys classify TDR as low (<5%), moderate (5%-15%) or high (>15%) in populations likely to have been recently infected [8]. Although rates of TDR remain low in most areas assessed using WHO-recommended methods [10], recent publications document moderate levels in specific geographic areas [11–14].

Phylogenetic analysis of protease and reverse transcriptase sequences used for the study of TDR allow the identification of transmission clusters (TC) and their correlation with transmission routes [15], drug resistance [16–17] and risk behavior [18–20]. The aims of this study are to carry out an epidemiological surveillance study on TDR among individuals newly diagnosed of HIV-1 infection through a nine year period in Spain and to assess the role of TC in the propagation of resistant strains.

## Materials and Methods

### Patients

This study includes 1614 antiretroviral drug-naive patients who were newly diagnosed of HIV-1 infection from January 2004 through December 2012 in 12 hospitals of the Public Health Service of two regions of Spain: Galicia and Basque Country. Epidemiological data of the patients are summarized in Table 1.

**Table 1:** Epidemiological characteristics of the study population and distribution of HIV-1 genetic forms.

	Total	Non-TDR	TDR	P value
Number of patients	1614	1453 (90.1%)	161 (9.9%)	
Gender				
Male	1262 (78.2%)	1132 (77.9%)	130 (80.7%)	0.4
Female	331 (20.5%)	301 (20.7%)	30 (18.7%)	0.5
Unknown	21 (1.3%)	20 (1.4%)	1 (0.6%)	0.4
Area of origin				
Spain	1087 (67.3%)	977 (67.2%)	110 (68.3%)	0.7
Latin America	210 (13%)	189 (13%)	21 (13.2%)	1
Africa	145 (9%)	134 (9.2%)	11 (6.8%)	0.3
Europe	68 (4.2%)	61 (4.2%)	7 (4.3%)	0.9
Unknown	104 (6.4%)	92 (6.3%)	12 (7.4%)	0.5
Transmission route				
Heterosexual contact	611 (37.8%)	557 (38.3%)	54 (33.5%)	0.2
Men who have sex with men	573 (35.5%)	512 (35.2%)	61 (37.9%)	0.5
Sexual	190 (11.8%)	172 (11.8%)	18 (11.2%)	0.8
Injecting drug use	149 (9.2%)	132 (9.1%)	17 (10.5%)	0.5
Other	13 (0.8%)	9 (0.6%)	4 (2.5%)	0.01
Unknown	78 (4.8%)	71 (4.9%)	7 (4.4%)	0.7
Subtype				
B	1142 (70.7%)	1012 (69.6%)	130 (80.7%)	0.003
Non-B genetic forms Subtypes:	472 (29.3%)	441 (30.3%)	31 (19.3%)	0.003
A	27		4	
C	52		6	
D	2		2	
F	84		1	
G	25		2	
H	1			
CRFs	174			
CRF01_AE	16			
CRF02_AG	95		2	
CRF06_cpx	4			
CRF11_cpx	2			
CRF12_BF	5		1	
CRF14_BG	8		4	
CRF19_cpx	1		1	
CRF20_cpx	1			
CRF22_01A	3			
CRF24_BG	4			
CRF26_AU	3		1	
CRF31_BC	4		2	
CRF43_02G	8		2	
CRF45_cpx	1			
CRF47_BF	19			
URFs	107		3	
Dual infections	5			

Most of the patients were men (78.2%). The average age was 37.1 years. One thousand and eighty seven (67.3%) were born in Spain and 423 (26.2%) were foreign-born, most of them from different countries of Latin America (13%) and Africa (9%). Transmission route was mainly through sexual contact, including 37.8% heterosexual individuals, 35.5% men who have sex with men (MSM), and 10.9% men who reported sexual transmission without specifying whether it was heterosexual or homosexual.

The study was approved by the Research Ethics and Animal Welfare Committee, Instituto de Salud Carlos III (CBBA/4 2008 PI 322 and CEI PI 51-2011-v2). Signed statements of informed consent were obtained from all patients.

### Viral RNA isolation, amplification, and sequencing

RNA was extracted from plasma samples with NucliSENS kit (bioMérieux, France), according to the manufacturer’s instructions. A *pol* region comprising HXB2 positions 2107 to 3630, containing the protease and partial reverse transcriptase (PR-RT) sequences, was amplified by reverse transcription-PCR, followed by nested PCR. Population sequencing was performed with ABI Prism BigDye Terminator Cycle Sequencing kit and ABI 3730 XL sequencer (Applied Biosystems, Foster City, CA, U.S.A.). Sequences were assembled with SeqMan 6.1 (DNASTAR, Madison, WI, USA), edited with BioEdit v.7.0 (http://www.mbio.ncsu.edu).

### Phylogenetic analyses

Sequences were aligned with MAFFT v.6 (http://mafft.cbrc.jp/alignment/software). Phylogenetic sequence analysis was performed via maximum likelihood (ML) using RAxML v.7 [21] applying the general time reversible substitution model with CAT approximation for among-site rate heterogeneity. Classification of sequences in subtypes and CRFs was based on clustering with clade references with a bootstrap value of ≥80% in the ML tree and on analyses with the online program COMET HIV-1 (http://comet.retrovirology.lu/). Sequences branching outside of clade reference clusters in the ML tree or suggested by the COMET analysis to be unique recombinant forms (URFs) were analyzed for recombination with Simplot v3.5 [22], using a 300 nucleotide window with tree construction by the neighbor-joining method applying Kimura’s two-parameter substitution model. TC were defined as those comprising viruses from five or more individuals whose sequences clustered in phylogenetic trees with a bootstrap value ≥90%. Sequences phylogenetically related to those from TC were searched in GenBank using the BLAST algorithm, with one sequence per cluster. The 10 database sequences with highest similarity scores for each TC sequence were subsequently included in phylogenetic analyses with RAxML. Database sequences clustering with TC from our study with ≥90% bootstrap values were incorporated into the TC.

### Analysis of antiretroviral drug resistance mutations

Resistance mutations to RT inhibitors (RTIs) and protease inhibitors (PIs) were analyzed according to mutations defined at the SDRM list updated in 2009 [9] through the Calibrated Population Resistance (CPR) tool version 6.0 available at Stanford University’s HIV Drug Resistance Database. (http://hiv-db.stanford.edu/pages/WHOResistanceList.html). TDR was defined as the detection of at least one mutation among the defined SDRM.

### Statistical analysis

Differences among categorical variables were analyzed by the chi-squared test with Yates’ correction, using the statistical program available at http://quantpsy.org/chisq/chisq.htm. P values <0.05 were considered significant.

## Results

Epidemiological data of the patients and distribution of HIV-1 genetic forms are summarized in Table 1. Overall, 1142 (70.7%) patients were infected with subtype B viruses and 472 (29.3%) with diverse non-subtype B genetic forms: 191 (11.8%) with 6 different subtypes, 174 (10.8%) with 15 CRFs, and 107 (6.7%) with URFs (Table 1).

The overall prevalence of TDR to any drug class among newly diagnosed HIV-1 infections during the 9 years of the study was 9.9% (161 of 1614). No statistically significant differences in the epidemiological characteristics were observed between patients with and without TDR. The frequency of TDR to any drug class among subtype B viruses was 11.2%, and among non-subtype B viruses it was 6.6% (p = 0.004).

**Fig 1:**
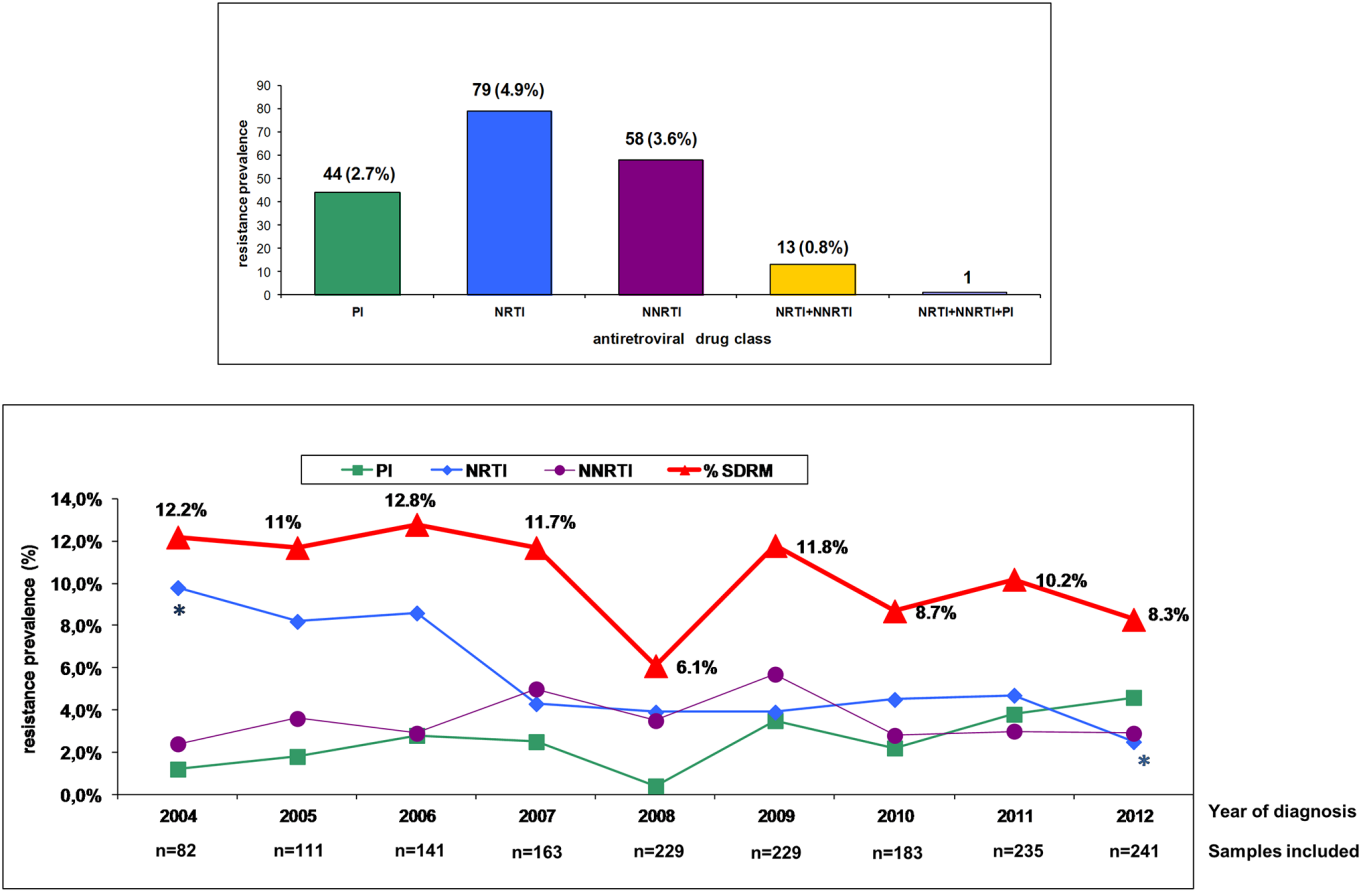
Prevalence and time trends of surveillance drug resistance mutations in antiretroviral drug-naive patients who were newly diagnosed of HIV-1 infection from January 2004 through December 2012 in 12 hospitals of the Public Health Service of two regions of Spain, Galicia and Basque Country. Asterisks indicate that the frequency of TDR to NRTIs from 10% in 2004 to 2% in 2012 was statistically significant (p = 0.01).

The prevalence of TDR according to the antiretroviral drug class involved in resistance was as follows: 4.9% to nucleoside RTIs (NRTIs), 3.6% to nonnucleoside RTIs (NNRTIs), 2.7% to PIs, 0.8% to NRTIs plus NNRTIs, and 0.06% to NRTIs plus NNRTIs plus PIs. Although there was a temporal trend for decreasing overall prevalence of TDR from 2004 (12.2%) to 2012 (8.3%), the difference was not statistically significant (p = 0.2), nor was it significant when only TDR to NNRTIs (p = 0.8) or to PIs (p = 0.3) were considered. However, the decrease in frequency of TDR to NRTIs from 10% in 2004 to 2% in 2012 was statistically significant (p = 0.01) (Fig 1).

The prevalence of mutations associated with resistance to NRTIs among the sequences with TDR is shown in Table 2.

**Table 2:** Prevalence of mutations in sequences with TDR.

NRTIs mutations		NNRTIs mutations		PIs mutations	
n = 79		n = 58		n = 44	
Mutation	N (%)	Mutation	N (%)	Mutation	N (%)
M41L	20 (25.3)	I85V	1 (1.7)	L23I	1 (2.4)
K65R	2 (2.5)	L100I	3 (5.1)	L24I	2 (4.8)
D67N	11 (13.9)	K101E	8 (13.8)	D30N	1 (2.4)
T69D	6 (7.6)	K103N/S	37 (63.8)	M46L/I	14 (33,3)
K70E	3 (3.8)	V106M	2 (2.5)	I54V/T	9 (21.4)
L74V	1 (1.3)	Y181C	7 (12.1)	D67G/N	2 (4.8)
Y115F	1 (1.3)	Y188L	5 (8.6)	G73S	2 (4.8)
M184V/I	8 (10.1)	G190A	5 (8.6)	L76V	2 (4.8)
L210W	14 (17.7)	P225H	2 (2.5)	V82A	8 (19)
T215C/E/S/V/D	48 (60.7)			N83D	1 (2.4)
T215Y	2 (2.5)			I84V	1 (2.4)
K219/Q/R/N/E	19 (24%)			N88D	1 (2.4)
				L90M	21 (47.7)

Considering the 79 sequences with TDR to NRTIs, thymidine analogue mutations (TAMs) were the most frequently detected, of which the most common were T215 revertants, present in 48 (60.7%) individuals, M41L in 20 (25.3%), K219Q/R/N/E in 19 (24%), L210W in 14 (17.7%), and D67N in 13.9%. The M184V/I major resistance mutation was detected in 8 (10.1%) of the 79 sequences. With regard to the 58 sequences with TDR to NNRTIs, the most frequent mutations were K103N/S (63.8%), K101E (13.8%), and Y181C (12.1%). Among the 44 sequences with TDR to PIs, the most common mutations were L90M (47.7%), M46L/I (33.3%), I54V/T (21.4%), and V82A (19%). It is noteworthy that several major mutations associated to high level resistance to NRTIs (K65R, L74V, Y115F, M184V and T215Y), to NNRTIs (l100I, K101E, K103N/S, V106M, Y181C, Y188L and G190A) and to PIs (D30N, M46I/L, I54V/T, L76V, V82A, I84V, N88D and L90M) were detected.

**Fig 2:**
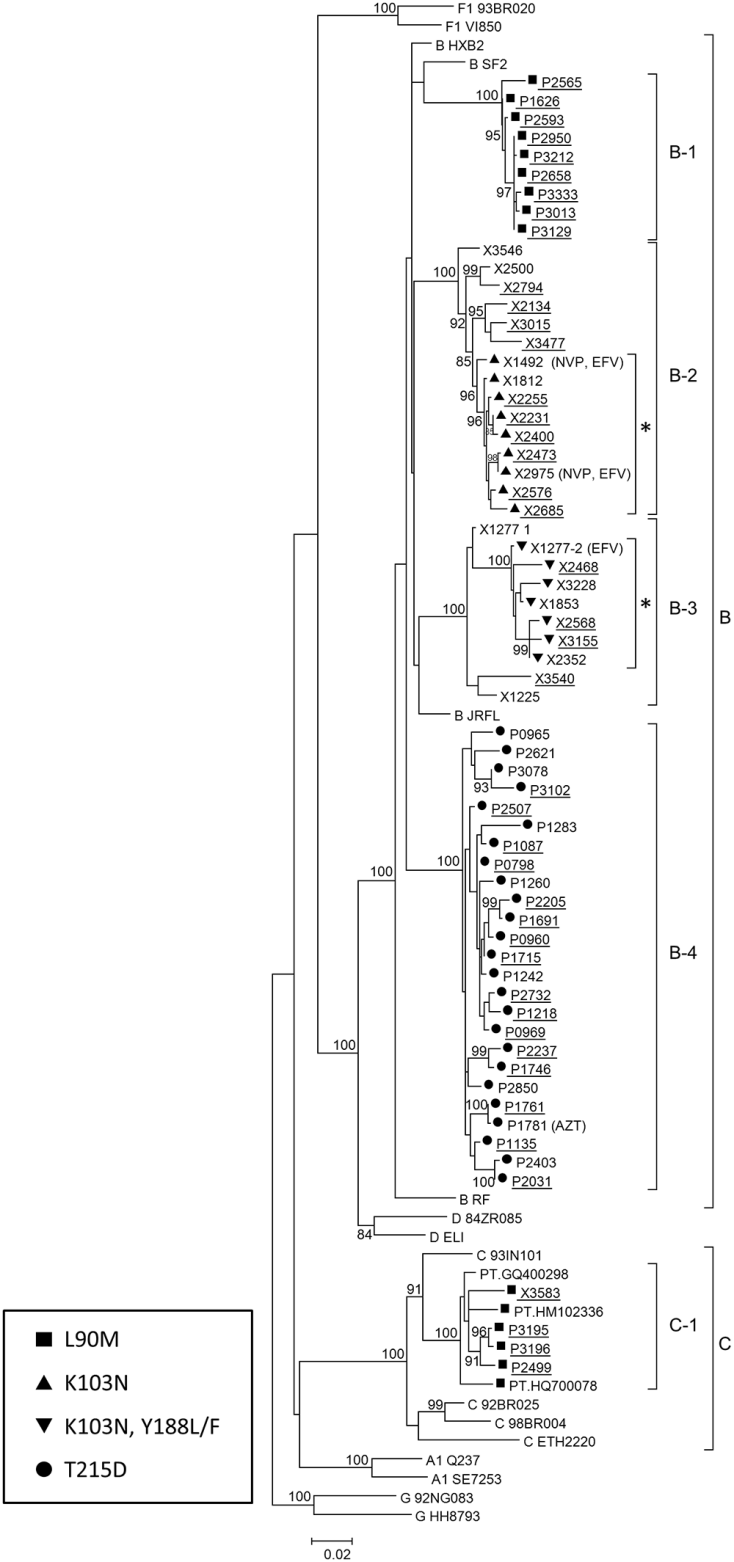
Maximum likelihood phylogenetic tree representing the five TC with TDR mutations, which are still growing, that is including newly diagnosed patients. Newly diagnosed HIV-1 infections are underlined. Antiretroviral drugs included in regimens of treated patients are indicated after each corresponding sample. Asterisks indicate subclusters with TDR which could have originated after antiretroviral treatment of patients X1492 and X1277. Sequences labeled PT followed by GenBank accessions represent viruses from Portugal retrieved from GenBank branching in C-1 cluster. Only bootstrap values ≥80% are shown.

The overall analysis of TC showed that 660 (40.9%) of the 1614 sequences of this study grouped in 120 clusters of five or more individuals. Sixty eight (42.2%) out of 161 sequences with TDR were involved in 25 TC. Among them, TDR mutations associated with high level resistance to antiretroviral drugs were detected in 9 clusters, 8 of subtype B and one of subtype C (named C4). Five of the 25 TC with TDR involved individuals diagnosed in 2012, 4 of them harboring high level resistance mutations (named B-1, B-2, B-3, and C-1) (Fig 2).

All the newly diagnosed individuals included in TC with TDR were men, and most of them (83.1%) were MSM. The L90M major mutation in protease, associated with high level resistance to atazanavir/ritonavir, indinavir/ritonavir, fosamprenavir/ritonavir, nelfinavir and saquinavir/ritonavir, was transmitted in clusters B-1 and C-1, which comprised 9 and 4 newly diagnosed individuals, respectively. Dates of diagnosis were between 2007 and 2012 (cluster B-1) and between 2009 and 2012 (cluster C-1). L90M was detected in all viruses of both clusters, except in one of three viruses from Portugal whose sequences were retrieved from GenBank clustering in C-1. Cluster B-2 comprised 15 individuals, 10 of them newly diagnosed between 2007 and 2012, in 6 of whom the K103N mutation, associated with high level resistance to first generation NNRTIs (nevirapine and efavirenz), was detected. This mutation was also observed in 3 other patients of this TC, 2 of whom were on treatment with nevirapine and efavirenz. All the 9 viruses harboring the K103N mutation grouped apart in a subcluster supported by a bootstrap value of 96%. Cluster B-3 comprised 4 newly diagnosed individuals with dates of diagnosis between 2008 and 2012, three of whom harbored the major mutations K103N and Y188L/F, associated with high level resistance to NNRTIs (nevirapine, efavirenz and rilpivirine). This mutation was also observed in other 4 patients of this TC, one of which had been treated with efavirenz. All 7 viruses harboring the mutations K103N and Y188L/F grouped apart in a subcluster supported by a bootstrap value of 100% (Fig 2).

Finally, all 25 individuals clustering in B-4 harbored the T215D revertant drug resistance mutation (Fig 2). Interestingly, 24 individuals were antiretroviral drug-naive and 16 of them were newly diagnosed of HIV-1 infection between 2004 and 2011. Twenty four of the 25 individuals clustering in B-4 reported sexual exposures, and only one was on treatment with zidovudine (ZDV)

## Discussion

This HIV-1 surveillance study has shown a moderate level (9.9%) of TDR to any drug class among 1614 newly HIV-1-diagnosed patients over 9 years. This frequency is similar to the prevalence of TDR detected in recent large studies conducted in other European countries, with an average frequency of 10% [2,13,14,23–26]. In Spain, the transmission of antiretroviral drug resistance has been studied within a large cohort of drug-naïve HIV-1-infected individuals (CoRIS) in two different periods of time, 2004–2008 and 2007–2010 [7,23], showing a prevalence of TDR of 8.5% in both studies and in agreement with the percentage observed in a study conducted in Madrid from 2000–2011 [27]

Individually, the first consequence of TDR mutations in newly diagnosed drug-naive patients is that the effectiveness of antiretroviral therapy is compromised when first line regimens include drugs to which HIV-1 has a reduced susceptibility, and consequently continuous prospective monitoring is indicated [23]. The prevalence of TDR regarding NRTIs (4.9%) and NNRTIs (3.6%) were close to the frequencies observed in the SPREAD study (4.7% and 2.3%, respectively) [23] and in previous studies in Spain, which were 4.4% and 4%, respectively, in 2004–2008 [7], and 3.92% and 3.82%, respectively, in 2007–2010 [28].

A trend towards decreasing prevalence of TDR over time from 12.2% in 2004 to 8.3% in 2012 was observed. The frequency of TDR associated with NRTIs decreased significantly from 10% in 2004 to 2% in 2012 (p = 0.01). This is primarily due to declines in 215 revertants and TAMs, which can be predicted based on the shift away from ZDV and d4T regimens. These results are similar to another previously reported study in Spain [7], but different from the results obtained in the Spread study where nucleoside resistance mutations remained constant at 5% throughout the study [29]

The prevalence of TDR in non-subtype B genetic forms was lower (6.6%) than in subtype B (11.2%) (p = 0.004). A lower prevalence of TDR in patients infected with non-subtype B viruses compared with those infected with subtype B viruses has been reported [30]. This could be in part due to the fact that the majority of patients carrying non-subtype B viruses would have likely acquired the infection in resource—limited countries in which treatment is only available to a minority of infected persons. It could be also explained by heterosexual transmission rather than MSM among non-subtype B infected patients. In contrast to the mentioned reports, a higher prevalence of TDR to NNRTIs was detected in non-subtype B viruses in Spain [6]. It is important to take into account that the International AIDS Society-USA (IAS-USA) list of TDR [31] was used in the Spanish study. In this regard, discrepancies between SDRM defined by WHO (2009) and by IAS-USA have been reported, showing a higher prevalence of TDR to NNRTIs using the IAS-USA list [32].

With regard to the transmission of resistant variants in TC, it was observed that 42% sequences with TDR were included in 25 TC of 5 or more individuals, underlining that 9 of them harbored TDR associated with high level drug resistance. Moreover, 4 of these 9 TC include patients diagnosed in the last two years. In previous studies, the contribution of TC to the spread of TDR has been reported at frequencies of 34.9% in newly diagnosed patients and of 52.7% in recent infections [33], and of 61.4% in antiretroviral drug-naïve individuals [17]. However, we defined TC as those comprising at least 5 individuals, while other studies included clusters with 2 or more individuals [25,27], with only 12.2% of clusters comprising 5 or more individuals in one of the previously published studies [29].

The most frequent TDR mutations to NRTIs were revertant mutations at 215 position (215rev) (60.7%), followed by M41L (25.3%) and K219Q/R/N/E (24%). This finding reflects the widespread usage of thymidine analogs in suboptimal regimens that led to the emergence and persistence of such variants. These figures are very similar to others reported previously [7,23,24,28]. It is probable that the high level of T215rev could be a late effect of the low efficacy of HAART regimens, or the use of mono or dual NRTI regimens with thymidine analogs. T215rev can revert back to the resistant codon 215Y/F rather efficiently under the selective pressure of ZDV [34], and consequently this antiretroviral drug would not be recommended as part of the antiretroviral treatment for patients harboring any of the 215rev mutations. M184V major mutation was detected in 10.1% of the sequences with TDR to NRTIs. It has been reported that the transmission of M184V has decreased since the 1990s, probably as a consequence of the use of more successful regimens.

In our study, one of the notable findings was the presence of a large cluster (B-4) involving 25 patients diagnosed between 2000 and 2011 harboring the T215D resistance mutation. This cluster corresponds to the expansion of a TC previously reported by us in 2009 [20]. Twenty four patients of the B-4 TC, including 16 newly diagnosed individuals, were antiretroviral drug-naïve, representing a sustainable reservoir of resistance to ZDV among new infections in patients with sexual risk exposures in the Basque Country. Infections belonging to this TC have been diagnosed along 11 years, indicating a continued circulation of viruses with this mutation, with the transmitter source dating to as early as 2000. In this regard, the transmission of T215S mutation within a cluster of newly diagnosed MSM for 2 years has been reported by other authors [25,33].

With regard to the TDR to NNRTIs, the most frequent mutations were K103N/S (63%), which is expected, since efavirenz and nevirapine have been part of the recommended first-line HAART regimens in Spain. Cluster B2 contains sequences with K103N variants as well as wild type. It is likely that K103N minority species may exist in these wild type infections. Future analysis using next generation sequencing would be important for hidden 103N variants that may perclude the use of EFV and NVP in first-line regimens. The major mutation K103N was detected in most sequences from newly diagnosed patients included in 2 TC, probably originated from patients failing therapy, with sustained circulation of viruses harboring this mutation for at least 7 years in one of these clusters. In this regard, a TC with multiclass drug-resistant viruses circulating among MSM for more than 2 years [35] has also been described. It should be noted that the genotypic resistance test applied in this study is population-based sequencing, with which only variants that make up more than 20% of the total viral population are detected. Therefore, it is foreseeable that TDR mutations involved in TC could be present as minority subpopulations in the remaining sequences, only detectable through ultra-deep sequencing or allele-specific PCR.

The major mutation L90M was the most common (45.2%) TDR to PIs. This mutation is mainly selected by saquinavir and nelfinavir. Although these antiretroviral drugs were frequently used in the late 1990s, their use has currently decreased. The high frequency of this mutation suggests continuing transmission among treatment-naïve newly diagnosed patients. It is also interesting to note that in two TC, one of subtype B and another of subtype C, the transmission of the major mutation L90M to PIs was observed in all the viruses of both TC during a period of at least 5 years, suggesting that the fitness of these viruses may play a role in their frequent transmission. MSM represented the most frequently reported risk exposure among these patients. In this regard, a high frequency of this mutation among antiretroviral drug-naïve MSM patients who grouped in a TC has been reported [33,36]. Recently, a large TC involving L90M mutation has been reported among antiretroviral drug-naïve MSM of the Swiss HIV Cohort Study with infection dates in 1996–2009, showing the continuous spread of this DRM even after the use of drugs selecting for them had dramatically decreased [17].

It has been described that transmission clustering is driven by primary/early stage infection [37]. This study argues for early diagnosis and treatment of persons harbouring TDR to avert the genesis of clustered outbreaks driving the spread of drug-resistant subepidemics, including viral lineages with 215 revertants, K103N/S and L90M, that are replicative fit [37].

In conclusion, this study provides data on epidemiological surveillance of TDR and their transmission in TC in Spain during a 9 year period, showing the relationship between local transmission clusters, the spread of resistant viruses and the overall prevalence of TDR mutations. The impact of epidemiological networks on TDR frequency may explain its persistence in newly diagnosed individuals and its knowledge may help clinicians in the selection of first antiretroviral drug regimens based on resistance screening. Besides this, the knowledge of the populations involved in TC would facilitate the design of prevention programs and public health interventions focusing on transmission chains, which could reduce the spread of TDR mutations.
